# Identification of Bioactive Phytochemicals in Mulberries

**DOI:** 10.3390/metabo10010007

**Published:** 2019-12-20

**Authors:** Gilda D’Urso, Jurriaan J. Mes, Paola Montoro, Robert D. Hall, Ric C.H. de Vos

**Affiliations:** 1 Department of Pharmacy, University of Salerno, 84084 Fisciano SA, Italy; gidurso@unisa.it (G.D.); pmontoro@unisa.it (P.M.); 2 Business Unit Fresh Food and Chains, Wageningen Food & Biobased Research, Wageningen University and Research, 6708 WG Wageningen, The Netherlands; jurriaan.mes@wur.nl; 3 Business Unit Bioscience, Wageningen Plant Research, Wageningen University and Research, 6708 PB Wageningen, The Netherlands; robert.hall@wur.nl; 4 Laboratory of Plant Physiology, Wageningen University and Research, 6708 PB Wageningen, The Netherlands

**Keywords:** mulberry, high resolution mass spectrometry, antioxidant activity, in vitro gastrointestinal digestion, α-glucosidase inhibitory activity

## Abstract

Mulberries are consumed either freshly or as processed fruits and are traditionally used to tackle several diseases, especially type II diabetes. Here, we investigated the metabolite compositions of ripe fruits of both white (*Morus alba*) and black (*Morus nigra*) mulberries, using reversed-phase HPLC coupled to high resolution mass spectrometry (LC-MS), and related these to their in vitro antioxidant and α-glucosidase inhibitory activities. Based on accurate masses, fragmentation data, UV/Vis light absorbance spectra and retention times, 35 metabolites, mainly comprising phenolic compounds and amino sugar acids, were identified. While the antioxidant activity was highest in *M. nigra*, the α-glucosidase inhibitory activities were similar between species. Both bioactivities were mostly resistant to in vitro gastrointestinal digestion. To identify the bioactive compounds, we combined LC-MS with 96-well-format fractionation followed by testing the individual fractions for α-glucosidase inhibition, while compounds responsible for the antioxidant activity were identified using HPLC with an online antioxidant detection system. We thus determined iminosugars and phenolic compounds in both *M. alba* and *M. nigra*, and anthocyanins in *M. nigra* as being the key α-glucosidase inhibitors, while anthocyanins in *M. nigra* and both phenylpropanoids and flavonols in *M. alba* were identified as key antioxidants in their ripe berries.

## 1. Introduction

Mulberry belongs to the genus *Morus*, plant family Moraceae, which comprises 24 different species and one subspecies, with at least 100 varieties [1]. The most commonly known *Morus* species are *Morus alba* (white mulberry), *Morus nigra* (black mulberry) and *Morus rubra* (red mulberry.) [2]. They are deciduous trees originating from China and Japan, and have spread into America and Europe for silkworm breeding. Globally, the main use of mulberry trees is to produce leaves as feed for cultivating silkworms, but in various regions, they are also much appreciated for their fruit, which can be consumed both fresh and as an ingredient in processed food products [3].

Mulberry species have been used in Traditional Chinese Medicines (TCM) for the treatment of several diseases, especially diabetes mellitus type II; they contain specific molecules, i.e., iminosugars or iminocyclitols, which are low molecular weight carbohydrates in which the endocyclic oxygen atom has been replaced by a nitrogen atom. These compounds are known to be able to inhibit the enzyme α-glucosidase which is present in the brush border of the human intestine [4,5,6]. Inhibition of α-glucosidase leads to a decreased rate of glucose absorption thus resulting in a lower postprandial blood glucose level. Inhibitors of α-glucosidase can prevent the development of diabetes in individuals with impaired glucose tolerance and/or impaired fasting of blood glucose [7].

Mulberries are also a good nutritional source of a variety of phenolic compounds, like flavonols and phenolic acids, as well as coloured anthocyanins in the case of black and red mulberry fruits [8,9,10]. Phenolic compounds are the subject of increasing scientific interest; they are natural antioxidants in plant-derived foods and food products and their intake is frequently related to human health. Many of the bioactivities ascribed to mulberries, such as antioxidant action, hypolipidemic effect and macrophage activating effect, have also been linked to their phenolic compound composition [11,12,13,14].

The present study aimed to determine differences in chemical composition between the ripe fruits of *M. alba* and *M. nigra,* which are the major species growing in Italy, by metabolomic analysis and to identify the bioactive compounds responsible for their antioxidant and α-glucosidase inhibitory bioactivities. To this purpose, we used I) HPLC-PDA with an online, post-column antioxidant detection system, and II) HPLC-PDA- HR Orbitrap FTMS with on-line fractionation into 96-wells plates followed by off line in vitro α-glucosidase inhibitory activity testing of the contents of the individual wells. A series of compounds present in mulberry fruits as well as major bioactives were further characterized using MS^n^ fragmentation. In addition, we subjected mulberries to an in vitro gastrointestinal digestion system in order to investigate potential effects of digestion on the observed bioactivities upon consumption of these berries.

## 2. Results

### 2.1. Identification of Phytochemicals in Morus using LC-PDA-Orbitrap FTMS

To identify the metabolites present in white and black mulberry fruits, HPLC-PDA-Orbitrap FTMS analysis was performed on their aqueous methanol extracts, thus generating both an LC-PDA and an LC-MS profile per extract (Figures S1 and S2, respectively). Based on the exact mass of their molecular [M+H]^+^ ion masses, their MS^n^ fragmentation patterns and their UV/Vis absorbance spectra, we putatively identified 35 compounds in the fruits (Table 1).

In the fruits of *M. nigra*, four anthocyanins with a characteristic absorbance maximum at around 500–520 nm were identified. Compound **6**, cyanidin hexoside, showed a pseudomolecular ion at *m*/*z* 449.1084, corresponding to the molecular formula C_21_H_21_O_11_^+^ that, upon fragmentation, gave one principal product ion at *m*/*z* 287.0546; Compound **8**, cyanidin hexose-deoxyhexose, showed a pseudomolecular ion at *m*/*z* 595.1662, corresponding to the molecular formula C_27_H_31_O_15_^+^, that fragmented into two principal product ions at 449.1058 (C_21_H_21_O_11_^+^: loss of deoxyhexose) and 287.0546 (loss of hexose+deoxyhexose); Compound **9**, pelargonidin hexoside, showed a pseudomolecular ion at *m*/*z* 433.1135, corresponding to the molecular formula C_21_H_21_O_10_^+^, and gave one principal product ion at 271.0596; Compound **11**, pelargonidin hexose-deoxyhexose showed a pseudomolecular ion at *m*/*z* 579.1714 corresponding to molecular formula C_27_H_31_O_14_^+^ that, upon fragmentation, gave two principal product ions of *m*/*z* 433.1115 and 271.0596. Thus, anthocyanins detected represented both pelargonidin and cyanidin conjugated with one, two or three C_6_-sugars. These anthocyanin compounds have previously been identified in *Morus alba* fruits [17] and are responsible for the dark color of black mulberry fruits. These anthocyanins were present in berries of *M. nigra* and were not detectable in those of *M. alba* (Supplemental Table S1), as was expected from their differential colors. 

A series of flavonols with different substituents were present in both white and black mulberry fruits (**12**, **14–17**, **19–24**, **26**, **28–30**, **32**, **35**). All these compounds showed the characteristic flavonol absorbance peaks at around 260 nm (resulting from the A-ring) and at around 350 nm (due to B-ring) and producing daughter ions of *m*/*z* 303.0496 (quercetin) or 287.0547 (kaempferol); many of the compounds detected have not previously been described for mulberry fruits. For instance, compounds **14**, **19**, **22** show the same pseudomolecular ion [M+H]^+^ at *m*/*z* 773.2135 with the same fragments of *m*/*z* 303.0496, 465.0995 and 611.1565, corresponding to the loss of two hexose and one deoxyhexose moiety, but with different RTs. These compounds were thus identified as different isomers of quercetin-hexose-hexose-deoxyhexose. A similar fragmentation pattern has been reported for a quercetin-trisaccharide in tomato fruit [19]. Specifically, compound **14** was confirmed as quercetin-3-O-rutinoside-7-O-glucoside based on the retention time of the reference compound reported in tomato fruit database [19].

Compounds **17**, **23**, and **24** likewise showed a similar pseudomolecular ion [M+H]^+^ at *m*/*z* 757.2192 with the same fragments of *m*/*z* 287.0547, 449.1065 and 611.1576, corresponding to the loss of two hexoses and one deoxyhexose moiety, but with different RTs. These were thus identified as kaempferol-hexose-hexose-deoxyhexose isomers. The fragmentation pattern of these compounds agrees with known kaempferol glycosides in tomato [19]. Moreover, compound **17** was confirmed as kaempferol-3-O-rutinoside-7-O-glucoside based on the retention time of the reference compound reported in tomato fruit database [19]. 

Compound **16** showed a pseudomolecular ion at *m*/*z* 713.1544 that upon fragmentation, gave three principal product ions at *m*/*z* 303.0496, 463.1021 and 551.1015 corresponding to the loss of two hexose moieties and one malonyl moiety; this compound was thus tentatively identified as quercetin hexose-malonyl-hexoside. This fragmentation pattern is consistent with that reported for a quercetin malonyl glucoside in lettuce [21]. Compound **21** showed a pseudomolecular ion at *m*/*z* 697.1597 which gave three principal product ions at *m*/*z* 287.0545, 449.1065 and 535.1076, corresponding to the loss of two hexose and one malonyl moiety; this compound was identified as kaempferol hexose-malonyl-hexoside. This compound showed a similar fragmentation pattern as reported in Cycorium intibus [24]. Thus, similar flavonol conjugates consisting of both quercetin and kaempferol esterified with one to three C_6_-sugars, or one or two sugars with one malonyl group, were present in both white and black mulberries. 

Compound **20** showed a pseudomolecular ion at *m*/*z* 451.1235 that gave one principal product ion at *m*/*z* 289.0703, corresponding to the loss of a hexose moiety; this compound was tentatively identified as dihydrokaempferol-hexoside. A similar fragmentation pattern was reported for dihydrokaempferol-hexoside in raspberry [23].

Three N-containing sugars, i.e., compounds (**1**) 1-deoxynojirimicin, (**2**) N-nonil deoxynojirimicin and (**3**) fagomine, were found in fruits of both mulberry species. These compounds have previously been reported for leaves of *M. alba* [16] and are well known for inhibiting the enzyme α-glucosidase and consequently, might contribute to an antihyperglycemic effect [4,5,6].

Four piperidine alkaloids, morusimic acids B, C and E (compounds **7**, **25**, **27**) were also identified based on their exact molecular mass and fragmentation; these compounds have previously been reported in fruits of *M. alba* from Turkey [18]. 

Both *M. alba* and *M. nigra* fruits also contained caffeoylquinic acids monomers (**5**, **10**, **13**) as well as dimers (**31**, **33**, **34**). All three caffeoylquinic acid isomers (**5**, **10**, **13**) showed a pseudomolecular ion at 355.1024 that, upon fragmentation, gave the same daughter ion at *m*/*z* 163.0386, corresponding to the loss of their quinic acid moiety. These compounds have also been reported in *M. alba* fruits from Serbia [17]. The three dicaffeoylquinic acid isomers (**31**, **33**, **34**) showed a pseudomolecular ion at 517.1341 that produced the same MS/MS base peak at 163.0387. These compounds have previously been reported in leaves of *M. alba* [27].

Compound **4** showed a pseudomolecular ion at *m*/*z* 182.0817, that fragmented into two principal product ions at 165.0544 and 136.0755. It was putatively identified as the alkaloid 2-formyl-1H-pyrrole-1-butanoic acid, previously reported in *M. alba* fruits by Kim et al. [11]. 

### 2.2. Global Metabolome Differences between Morus Alba and Morus Nigra Fruits

Ripe fruits of *Morus alba* and *Morus nigra* were collected from trees growing at various locations in the Campania Region (Italy) and subjected to untargeted LCMS-based metabolite profiling. Subsequent unbiased data processing generated a dataset with the relative intensities and in-source mass spectra of 361 putative metabolites in the samples (Supplemental Table S1; note that this metabolite list misses some of the manually identified compounds described in Table 1, indicating that one or more parameter settings in the untargeted data processing workflow appears suboptimal for these specific compounds). An unsupervised multivariate statistical method, Principal Components Analysis (PCA), was subsequently applied to the entire metabolite dataset resulting in a clear differentiation of *M. alba* and *M. nigra* fruit samples (Figure S3). Among the most significantly (*p* < 0.05) differing metabolites were anthocyanins (Supplemental Table S1), as was expected from the differential fruit colours of both species. In addition, it was possible to identify two other flavonoids only detectable in *M. nigra*, namely dihydroquercetin hexoside and dihydrokaempferol hexoside. In fact, an important step for the biosynthesis of anthocyanidins is the reduction of dihydroflavonols catalysed by the enzyme DFR (dihydroflavonol 4-reductase) converting dihydroquercetin and dihydrokaempferol into colorless leucoanthocyanidins, which are further converted by the enzyme anthocyanin synthase (ANS) into cyanidin and pelargonidin, respectively [29], thereby providing the fruit colour in *M. nigra*. Several flavonol conjugates, including quercetin glycosides **19** and **26** and kaempferol glycoside **29** (Table 1), were also significantly (*p* < 0.05) higher in *M. nigra* fruit, while the mono- and di-caffeoyl quinic acids (phenylpropanoids) were not differential between both fruit species (Supplemental Table S1). These data suggest that *M. nigra* fruits exhibit a higher activity of the general flavonoid pathway than *M. alba* fruit. The alkaloids identified did not significantly differ between the *M. nigra* and *M. alba* fruit samples analyzed (Supplemental Table S1). 

### 2.3. α-Glucosidase Inhibitory Activity and Effect of In Vitro Gastrointestinal Digestion

**Figure 1:**
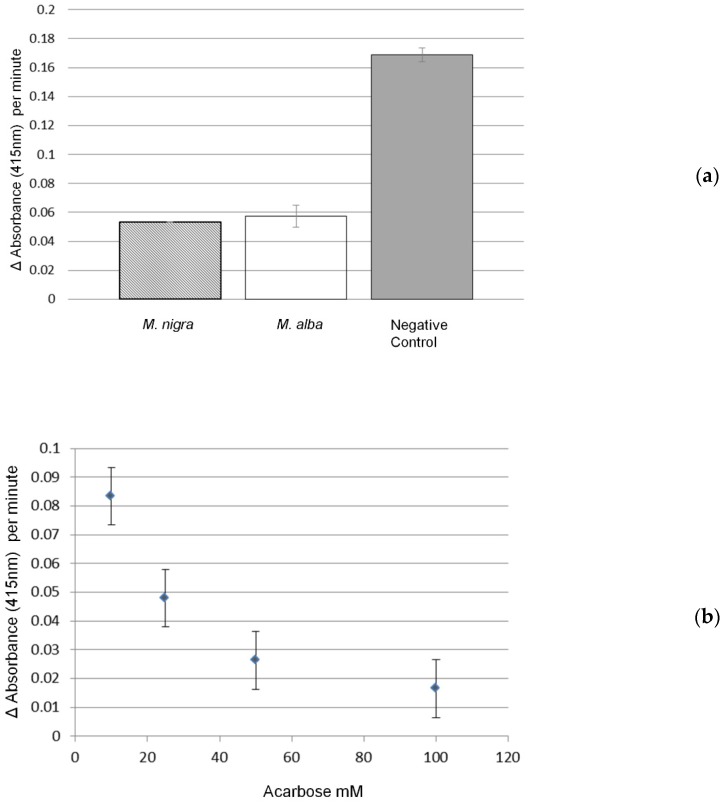
α-glucosidase inhibitory activity of mulberry methanol extracts. The Y axis represents the α-glucosidase activity (increase in 415 nm absorbance per minute) and the X axis the sample type tested. (**a**) inhibitory activity of water extracts of *Morus alba* and *Morus nigra* compared to the negative control (water). (**b**) enzyme activity inhibition by acarbose (positive control) at increasing concentrations (mM) in the assay. Data represent means and standard deviations (*n* = 3 assays).

The α-glucosidase inhibitory activity of the black and white mulberries was firstly evaluated using the crude aqueous-methanol extracts of the fresh fruits. The extraction solvent was evaporated by freeze-drying and the metabolites re-dissolved in MQ-water. These water extracts were then tested for inhibiting α-glucosidase enzyme activity, monitored through the increase in the pNP product, detected at 412 nm, using 96-wells plates kept at 30 °C; the α-glucosidase inhibitor acarbose was used as a positive control (Figure 1b). Both mulberry extracts showed a marked and similar α-glucosidase inhibitory activity as compared to the water blank (Figure 1a).

The *M. nigra* fruit showed an IC_50_ value of 0.75 ± 0.004 mg/g DW (*n* = 3), while that for *M. alba* fruit was 0.93 ± 0.003 mg/g DW (*n* = 3). In comparison, the IC_50_ value of acarbose was 13.83 ± 0.02 mg/g.

**Figure 2:**
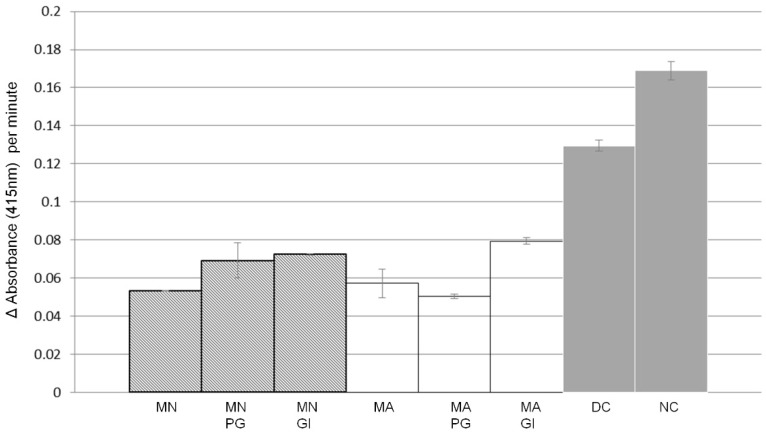
α-glucosidase inhibitory activity after in vitro gastrointestinal digestion. Inhibition activity of original *Morus nigra* (MN) and *Morus alba* (MA) fruit extracts, and after their in vitro stomach (Post Gastric, PG) digestion and in vitro gastrointestinal (GI) digestion. DC: digestion control, representing the digestion process, including all enzymes, without plant material; NC: negative control (NC), representing only water. Data represent means values and standard deviations (*n* = 3 measurements).

A simulated gastrointestinal digestion was then applied to estimate the effect of consumption and digestion on the α-glucosidase inhibitory activity of mulberry fruits (Figure 2). For both fruit types, the bioactivity measured in the original fruit extracts was partially lost upon this in vitro digestion (GI samples compared to MN and MA samples). Gastric digestion (PG) resulted in a slight decrease in bioactivity in *M. nigra* only. The control incubation consisting of water instead of fruit extract in the digestion test (DC samples) showed a slight inhibition of the α-glucosidase activity as compared to the negative control (NC of undigested fruits: water instead of both fruit extract and digestion enzymes and buffer): a decrease of 0.04 enzyme units. Taking this inhibiting effect of the digestion conditions on α-glucosidase into account, it was calculated that the simulated gastrointestinal digestion resulted in an overall reduction in α-glucosidase inhibitory activity of about 50% (a decrease of about 0.055 units from 0.13 in DC to 0.075–0.08 in GI samples, compared to a decrease of about 0.115, i.e., from 0.17 units in NC to about 0.055 in original MN and MA extracts). 

### 2.4. LCMS Combined with 96-Well Format Fractionation

In order to pinpoint those compounds in *Morus* fruits that are responsible for the observed α-glucosidase inhibitory activity, we subsequently used HPLC separation combined with both 96-well plate fractionation and Orbitrap FTMS detection. Injection, fractionation and FTMS analyses of the *M. alba* and *M. nigra* crude extracts, as used in the α-glucosidase inhibition assay, were performed in triplicate; a water blank was injected as a control. The fractionation plates were subsequently dried under a gentle N_2_ flow at 30 °C, the dried well contents re-dissolved in water and tested for α-glucosidase inhibitory activity. Compounds present in bioactive wells were then further characterized from their corresponding UV/Vis spectra and FT-MS data.

**Figure 3:**
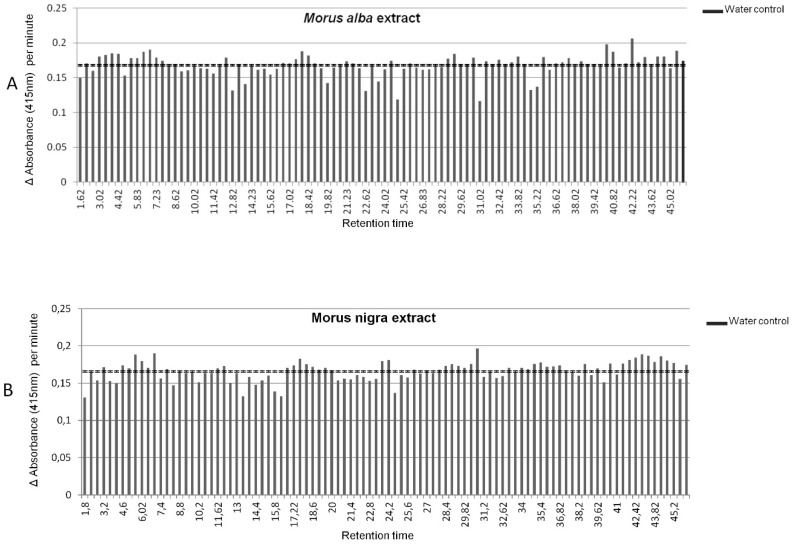
α-glucosidase inhibitory activity of 96-well LC-MS fractions of (**A**) *M. alba* and (**B**) *M. nigra* extracts. The Y axis shows the enzyme activity and the X axis the retention time corresponding to the LC-MS fraction. The vertical line at an enzyme activity of 0.17 indicates the average value in the water control. The wells considered bioactive are the ones below an enzyme activity value of 0.15.

The results of the α-glucosidase inhibitory activity of individual wells are shown in Figure 3A,B for *M. alba* and *M. nigra*, respectively. Based on the average enzyme activity measured in the wells of the water control sample, we set a threshold value at 0.17 absorbance units per minute, below which we considered a sample well to possess α-glucosidase inhibitory bioactivity. 

The compounds identified in the active wells of both fruit types were the amino sugar acids **1–3** and the flavonoids **15**–**17**, **19**–**20** and **32**. Two anthocyanins, **6** and **9**, only present in *M. nigra* (see Table 1), also showed bioactivity. In addition, other fractions clearly showing α-glucosidase inhibitory were detected e.g., between 34.7 and 35.2 min in *M. alba*, although we have yet been unable to pinpoint and identify the specific bioactive compound(s) (Table 2).

### 2.5. Total Antioxidant Activity and HPLC with Online Antioxidant Detection

The total antioxidant activity of the mulberry fruits was compared between other fruits well known for their antioxidant activity: cultivated strawberry (*Fragaria* × *ananassa*) and wild strawberry (*Fragaria vesca*). This antioxidant assay (Table 3) indicated that the aqueous-methanol extract of *M. nigra* is slightly more active than that of *M. alba*; in fact the mulberry fruits showed about the same activity as strawberry, which is among the fruit species with the highest antioxidant capacity [30]. 

**Figure 4:**
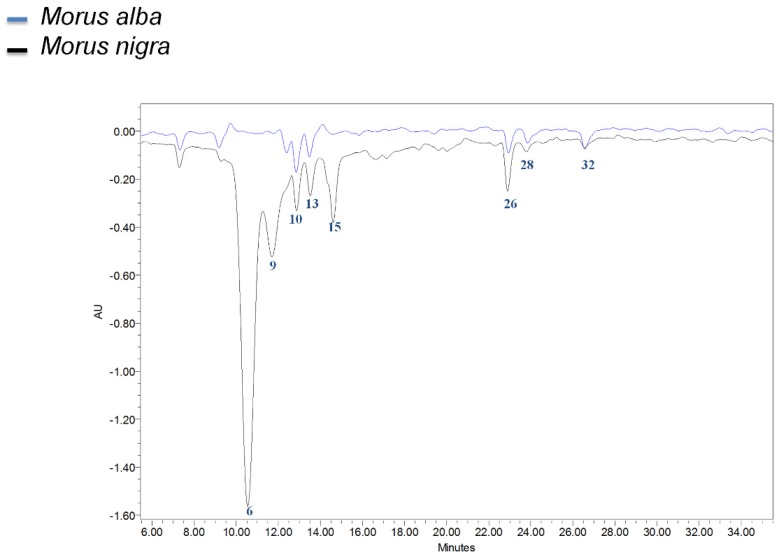
Antioxidant activity. Overlay of representative antioxidant chromatograms of fruit of *Morus alba* (in blue) and *Morus nigra* (in black). Antioxidant profiles of fruit extracts were determined online, by a post column reaction with ABTS^·+^ cation radicals after HPLC separation and PDA detection of compounds. The ABTS-radicals remaining after post-column reaction were recorded at 600 nm: negative peaks thus indicate antioxidant activity. The numbers refer to the main peaks identified (see Table 1): **6** cyanidin hexoside, **9** pelargonidin hexoside, **10** and **13** caffeoylquinic acid isomers, **15** dihydroquercetin hexoside, **26** quercetin hexose deoxyhexose, **28** quercetin hexoside, and **32** kaempferol hexoside.

Subsequently, a HPLC-PDA system coupled to online ABTS^·+^ cation radical reaction and detection [31] was used to determine the relative contribution of each individual component to the total antioxidant activity (Figure 4). Several antioxidant components could be identified by comparison of their retention times and absorption spectra with those of the LC-PDA-FTMS/MS analysis using the same chromatographic conditions. According to this online antioxidant assay, the key antioxidants in *M. nigra* corresponded to anthocyanins, in particular, compounds **6** and **9**. The other compounds responsible for antioxidant activity in both *M. alba* and *M. nigra* were caffeoylquinic acids, like compounds **10** and **13**, and flavonols like compounds **15**, **26**, **28**, **32** (see Table 1).

## 3. Discussion

In the present study, we compared ripe fruits of *Morus alba* and *Morus nigra* for their metabolite composition in relation to their potential relevant bioactivities upon consumption, i.e., α-glucosidase inhibiting and antioxidative activities. Using HPLC-PDA-Orbitrap FTMS analysis of aqueous-methanol extracts, we were able to detect a large series of compounds and identified a number of metabolites, previously reported for mulberry or other fruit species, as well as new compounds being present in either or both *M. alba* and *M. nigra*. Fruit of both species exhibited a marked α-glucosidase inhibiting activity in vitro, an indication of their potential beneficial effect with regard to type II diabetes. Moreover, we showed that this α-glucosidase inhibiting activity was partially resistant to simulated gastric and intestinal digestion. Anthocyanins appear among the potential bioactive compounds in *M. nigra* fruit (Figure 3) and the general instability of anthocyanins at the alkaline conditions of gastrointestinal digestion [32] may at least partly explain the loss of α-glucosidase inhibitory activity in *M. nigra* fruits. When calculating the α-glucosidase inhibiting activity of mulberries in units of acarbose, a well known type II diabetes drug based on its α-glucosidase inhibiting activity (https://www.drugs.com/pro/precose.html), our data suggest that consumption of about 20–25 g of fresh mulberry fruit corresponds to 50 mg of acarbose, taking into account a 50% bioactivity loss upon digestion. It has been shown that an intake of 100 mg acarbose 3 times a day can significantly reduce type II diabetes risk factors [33]. Thus, a daily consumption of 100–150 g fresh mulberries may exert relevant pharmacological effects with regard to type II diabetes. Using analytical LC-based extract fractionation, it was possible to pinpoint three known iminosugar acids, i.e., [1-deoxynojirimycin (**1**), N-nonil-deoxynojirimycin (**2**) and fagomine (**3**)], and 7 phenolic compounds, including five flavonols [dihydroquercetin hexoside, (**15**) quercetin hexoside malonyl hexoside (**16**), kaempferol-3-O-rutinoside-7-O-glucoside (**17**), quercetin hexose (**28**) and kaempferol hexoside (**32**)] present in both *M. alba* and *M. nigra*, and 2 anthocyanins [cyanidin hexoside (**6**), pelargonidin hexoside (**9**)] only present in *M. nigra*, as the key α-glucosidase inhibitors in mulberry fruits. While compounds **1**, **2**, **3**, **6**, **28** and **32** have already been reported to exert this bioactivity [5,16,34,35], in our study, we were able to detect novel α-glucosidase inhibitory compounds in mulberries. A similar approach, using accurate mass LCMS coupled to 96-well fractionation and bioactivity testing, has recently been used to identify novel compounds in pepper fruits interacting with the human hot-taste receptor [36].

Although it was not yet possible to identify the novel α-glucosidase inhibitory compounds in mulberry fruits on the basis of the observed accurate mass only, this method can well be optimized and adapted for further structural characterization of these bioactives, e.g., by using so-called multistage mass spectrometry at high mass resolution [19], if needed, combined with NMR experiments. For the latter approach, the same bioactive wells from replicate plates may be pooled to get sufficient NMR signals for the de novo identification. Alternatively, bioactive extracts can be re-injected in a LC-MS-SPE set up to collect and concentrate individual LC-MS peaks upon repeated injections; the SPE cartridges containing the active compounds (based on their known accurate mass and LC-retention time) can then be subjected to NMR for structural elucidation [37]. 

In addition to the α-glucosidase inhibitory activity, the ABTS^+^-radical based total antioxidant assay indicated significant antioxidant activity present in the same mulberries, comparable to that of strawberries, which are among the fruit species with the highest antioxidant capacity [32]. The higher activity in *M. nigra* compared to *M. alba* fruits is likely due to the presence of anthocyanins, which both provide fruits with their dark color and contribute to antioxidant activity [38]. Indeed, using HPLC with online antioxidant detection [32], we were able to pinpoint anthocyanins as the main phenolic antioxidants in *M. nigra*, while both phenylpropanoids and flavonols were the key phenolic antioxidants in *M. alba*. 

This work shows that it is well possible, using analytical scale techniques, to pinpoint the compounds that are key to the well described bioactivities of mulberry fruits, and to validate the value of metabolomics technologies in the phytochemical and bioactivity evaluation of functional foods. However, further studies towards, for example growth conditions, genotypic variation, fruit development and ripening are needed to obtain the best material for preparation of such functional foods with optimal composition of bioactive ingredients or for purification of the bioactive compounds.

## 4. Materials and Methods

### 4.1. Mulberry Materials

Fruits of *M. alba* and *M. nigra* were manually picked at ripe stage in May 2014 in different areas of the Campania region in Italy, in particular, the geographical locations Solofra (GPS coordinates latitude: 40.8291: longitude: 14.8456), Roccadaspide (GPS coordinates latitude: 40.4253: longitude: 15.1917), Fisciano (GPS coordinates latitude: 40.7728: longitude: 14.7994), San Sossio Baronia (GPS coordinates latitude: 410712: longitude: 15.2005). *Morus alba* fruits (MAF) were collected from 4 locations and coded as MAF-S (collected in Solofra) MAF-big (collected in San Sossio Baronia), MAF-wt (collected in San Sossio Baronia), MAF-R (collected in Roccadaspide); *M. nigra* fruits (MNF) were collected at five locations and coded as MNF-R (collected in Roccadaspide), MNF-U14 (collected in Fisciano), MNF-U13 (collected in Fisciano), MNF-S (collected in Solofra) and MNF-U13 (collected in Fisciano). All samples were botanically identified by Prof. V. De Feo (Department of Pharmacy University of Salerno) and compared with reference materials, then were freeze-dried before being transported to The Netherlands. They were then ground to a fine powder and stored at −80 °C until analysis.

For comparing antioxidant activities, fresh frozen mulberries were compared with fresh fruit of strawberry and wild strawberry collected in June 2014 in Campania (Italy).

### 4.2. Extract Preparation

The sample extracts used for LC-MS analysis were prepared essentially as described in De Vos et al. [31]: 30 mg of freeze dried samples were extracted with 1200 µL of 75% methanol in MQ water containing 0.1% of formic acid. Mixtures were then sonicated for 15 min, centrifuged at 12,500 g for 10 min and filtered over a 0.45 µm filter (Minisart SRP4, Biotech GmbH, Germany).

For α-glucosidase inhibitory activity testing, for both NanoMate fractionation and HPLC with online antioxidant analysis, 1 mL of supernatant was dried in a speedvac and taken up in 250 µL of water, sonicated and filtered through a 0.45 µm filter (Minisart SRP4, Biotech GmbH, Germany). These concentrated extracts were prepared in 3 independent replicates.

### 4.3. LC-PDA-Orbitrap FTMS Analysis

A metabolite analysis was performed using an HPLC (Waters Aquity) coupled to both a photodiode array detector (PDA; Waters) and an LTQ Ion trap-Orbitrap Fourier transformed Mass spectrometer (FTMS; Thermo) hybrid system. A Luna 3 µm C18 150 × 2 mm column (Phenomenex, USA) at 40 °C was used to separate the extracted metabolites, with MQ water with 0.1% formic acid (A) and acetonitrile with 0.1% formic acid (B) as solvents. A linear gradient from 5% to 35% B in 45 min, at a flow rate of 0.19 mL/min, was used [33]. In order to prevent possible order and batch effects in the LCMS analysis, all samples were analysed in a single series and in random order. Three quality control samples (QCs) prepared from a mix of all samples were included and equally distributed over the study samples, in order to check system stability and estimate overall technical variation.

Electrospray ionization (ESI) in positive mode was used to generate ions from eluting compounds. ESI source parameters were as follows: capillary voltage 43 V; tube lens voltage 120 V; capillary temperature 295 °C; Sheath and Auxiliary Gas flow at 40 and 3 (arbitrary units), respectively, Sweep gas 0 n, Spray voltage 5 V. MS spectra were acquired by full range acquisition covering *m/z* 104–1350 at a resolution of 60,000 FWHM.

### 4.4. LCMS data Processing and Multivariate Analysis

The raw LCMS data files were processed using Metalign software [34] for baseline correction, noise estimation, and ion-wise mass spectral alignment. MSClust software [35] was then used to assemble redundant mass signals derived from the same metabolite, including natural isotopes, adducts and in-source fragments, based on their corresponding retention times and relative abundance patterns across samples. This resulted in the relative intensities of 361 mass peak clusters, each representing a (reconstructed) putative metabolite, present in at least two samples. These metabolite intensity data were then subjected to a multivariate analysis using GeneMaths XT software version 2.12 (Applied Maths, Belgium). Metabolite intensities were firstly log2-transformed and then mean-centred across samples.

Using multiple online databases, including KNApSAcK (http://kanaya.naist.jp/KNApSAcK/), Dictionary of Natural Products (http://dnp.chemnetbase.com), Metlin (https://metlin.scripps.edu/), HMD (http://www.hmdb.ca), in-house libraries based on standards, as well as the mass spectra information within the clustered mass peaks and from additional LC-MS^n^ runs generating accurate mass spectral trees from the top 3 intensity ions every 30 s [19]. Selected metabolites (Table 1) were manually annotated as far as was possible using the mass data and the UV/Vis-absorbance spectral data available.

### 4.5. In Vitro Simulated Gastrointestinal Digestion

In vitro digestion was carried out on freeze-dried fruit samples of both *M. alba* and *M. nigra*, following the protocol described by McDougall et al. [36] with slight modification. Release of phytochemicals from fruit was checked by LC-MS at different stages of digestion, i.e., after gastric digestion (post gastric, PG) and gastrointestinal digestion (GI). Both PG and GI samples were stored at −80 °C until further analysis. During the process, three different controls were used: (1) plant material without digestion solutions and enzymes, diluted in water using the same ratio used for the samples coming from the digestion process (2) the solutions with all the ingredients for digestion but without plant material (DC: Digestion control), (3) plant material with all the ingredients for digestion but without active enzymes (the enzymes where added at the end of the digestion process, to the cold extract). Both α-glucosidase inhibitory activity and antioxidant activity were investigated for each of these PG and GI samples using the methods described below. 

### 4.6. α-Glucosidase Inhibition Assay 

The α-glucosidase assay uses the synthetic substrate p-nitrophenyl-α-D-glucopyranoside (pNPG), which is hydrolyzed by α-glucosidase to release p-nitrophenol (pNP), a color agent that can be monitored at 415 nm. Briefly, 10 µL of extract was combined with 40 µL of 100 mM phosphate buffer (pH 6.8) and 20 µL of α-glucosidase (0.6 units per mL buffer). After mixing and incubation for 5 min at 37 °C, 20 µL of a 20 mM pNPG solution in buffer was added to start the reaction. The reaction was monitored in time at 415 nm by a TECAN SpectraFluor microplate reader. Acarbose was used as a positive control, while water was used as a negative control for enzyme inhibition. The enzyme activities were evaluated as increase in the absorbance at 415 nm per minute and the percentage of enzyme inhibition was calculated. Three dilution series of extracts were used for IC_50_ determination. Dose−response curves and IC_50_ values were obtained by use of GraphPad Prism (version 6.00.283). The assay was performed with 3 replicates. 

### 4.7. NanoMate LC-Fractionation of Extracts

The HPLC–PDA–FTMS system was adapted with a chip-based nano-electrospray ionization source/fractionation robot (NanoMate Triversa, Advion BioSciences) coupled between the PDA and the inlet of the Ion Trap/Orbitrap hybrid instrument [19]. In this system, the compounds separated and eluting from the analytical column firstly passed the PDA detector for determining their UV/Vis absorbance spectra and then the eluent was automatically split by a NanoMate LC-fraction collector/injection robot (Advion) into a nanoflow for chip-based ESI nanospray Orbitrap FTMS analysis and the rest for fractionation into microwells with a collection time of 28 sec per well. The sample injection volume was 5 µL. The gradient and flow conditions were the same as described above, with an additional 30 µL/min 100% isopropanol added into the LC flow via a T-junction between the PDA and the NanoMate, in order to improve the solvent composition for generating a stable nano-electrospray. The eluent flow was split by the NanoMate at a ratio of 219.5 µL/min to the fraction collector and 0.5 µL/min to the nano-electrospray source. LC-fractions were collected every 28.2 s (i.e., 100 µL solvent) into 96-well plates (Twin tec, Eppendorf). After collection, the plates were dried at 30 °C under a gentle N_2_ flow, and then tested for α-glucosidase inhibitory activity as described above (performed in 3 replicates). 

### 4.8. Antioxidant Activity and HPLC Analysis with Online Antioxidant Detection

The total antioxidant capacity of fruits was analyzed using the ABTS^·+^ radical scavenging method, essentially according to Capanoglu et al. [37] with slight modifications. The fruits were collected in June 2014 and the antioxidant activity was tested on basis of fresh weight. 0.5 g of samples (fresh fruit) were extracted in 1.5 mL of methanol (final MeOH concentration about 77%, taking into account a fruit water content of 95%) containing 0.05% of formic acid, sonicated for 15 min and centrifuged at 12,500 g for 15 min, filtered through 0.45 µm (Minisart SRP4, Biotech GmbH, Germany) and then 10 µL of extract was used to test the antioxidant activity. Trolox was used as a reference.

To determine total antioxidant capacity, 10 µL of sample extracts or standard solution was mixed with 90 µL of ABTS-radical working solution (pH 7.4) and after 40 s, the remaining ABTS^·+^ radicals were measured at 415 nm using 96-well microplates (Nunc, Roskilde, Denmark) and an Infinite^®^ M200 micro plate reader (Tecan, Gröding, Austria). The analyses were done using 3 replicates and the results were expressed in terms of mg Trolox Equivalent Antioxidant Capacity (TEAC) per g fruit FW. In addition, the contribution of individual antioxidants to the total antioxidant capacity of the crude mulberry extracts was determined using an HPLC-PDA system coupled to post-column on-line antioxidant detection [37,38]. For this, the extracts of *M. alba* and *M. nigra* fruits, also used for the LC-MS analysis, were analyzed using a W600 Waters HPLC system coupled to a Waters 996 PDA detector (240–600 nm) [37,38]. Eluted compounds were allowed to react online for 30 s at 40 °C in a buffered solution of ABTS·^+^ cation radicals (pH 7.4). Then, the absorption of the remaining ABTS^·+^ radicals was monitored at 412 nm by a second detector (Waters 2487, dual-wavelength UV–vis detector). Peak identification was done by comparing PDA-absorbance spectra and retention times of eluting peaks with data taken from the literature and annotations were confirmed by HPLC-FTMS and MS/MS analyses, as described above.

## Figures and Tables

**Table 1:** Metabolites manually identified in *Morus alba* and *Morus nigra* using accurate mass LC-MS^n^ in positive ESI mode. L. I.: level of identification according to the Metabolomics Society Initiative [15].

N°	RT	Accurate Mass	Molecular Ion [M+H]^+^	Molecular Formula	Putative ID	Fragment Ions [M+H]^+^	L. I.	References
**1**	1.58	163.0844	164.0923	C_6_H_13_NO_4_	1-deoxynojirimycin	-	3	[16]
**2**	1.70	289.2253	290.2332	C_15_H_31_NO_4_	n-nonil-deoxynojirimycin	206.8857/122.9243	3	[16]
**3**	2.14	147.0895	148.0974	C_6_H_13_NO_3_	fagomine	-	3	[16]
**4**	2.19	181.0738	182.0817	C_9_H_11_NO_3_	2-formyl-1H-pyrrole-1-butanoic acid	165.0544/136.0755	3	[11]
**5**	8.16	354.0951	355.1024	C_16_H_18_O_9_	caffeoylquinic acid isomer I	163.0386	2	[17]
**6**	9.30	449.1084	449.1084	C_21_H_21_O_11_^+^	cyanidin hexoside	287.0546	2	[17]
**7**	9.35	507.3043	508.3122	C_24_H_45_NO_10_	morusimic acid E	346.2587/284.2579	3	[18]
**8**	10.21	595.1662	595.1662	C_27_H_30_O_15_^+^	cyanidin hexose deoxyhexose	449.1058/287.0546	2	[17]
**9**	10.93	433.1135	433.1135	C_21_H_21_O_10_^+^	pelargonidin hexoside	271.0596	2	[17]
**10**	11.52	354.0951	355.1024	C_16_H_18_O_9_	caffeoylquinic acid isomer II	163.0386	2	[17]
**11**	11.92	579.1714	579.1714	C_27_H_31_O_14_^+^	pelargonidin hexose deoxyhexose	433.1115/271.0596	2	[17]
**12**	12.24	626.1483	627.1542	C_27_H_30_O_17_	quercetin hexose hexose	465.1023/303.0489	2	[17]
**13**	12.38	354.0951	355.1024	C_16_H_18_O_9_	caffeoylquinic acid isomer III	163.0386	2	[17]
**14**	12.42	772.2062	773.2135	C_33_H_40_O_21_	quercetin-3-*O*-rutinoside-7-*O*-glucoside	303.0496/465.0995/611.1576	1	[19]
**15**	13.25	466.1111	467.1190	C_21_H_22_O_12_	dihydroquercetin hexoside/taxifolin hexoside	449.1069/305.0650	3	[20]
**16**	14.41	712.1487	713.1544	C_30_H_32_O_20_	quercetin hexoside malonyl hexoside	551.1015/463.1021/303.0496	2	[21]
**17**	14.71	756.2112	757.2192	C_33_H_40_O_21_	kaempferol-3-*O*-rutinoside-7-*O*-glucoside	611.1576/449.1065/287.547	1	[19]
**18**	15.35	386.1940	387.2020	C_19_H_30_O_8_	roseoside	370.1118/208.0599	3	[22]
**19**	16.37	772.2062	773.2135	C_33_H_40_O_21_	quercetin-hexose-hexose-deoxyhexose	303.0496/465.0995/611.1576	3	[19]
**20**	16.52	450.1162	451.1235	C_21_H_22_O_11_	dihydrokaempferol-hexoside	289.0703	3	[23]
**21**	17.02	696.1517	697.1597	C_30_H_32_O_19_	kaempferol hexoside malonyl hexoside	287.0545/449.1065/535.1076	2	[24]
**22**	17.22	772.2062	773.2135	C_34_H_40_O_31_	quercetin-hexose-hexose-deoxyhexose	303.0496/465.0995/611.1576	2	[19]
**23**	18.28	756.2112	757.2192	C_33_H_40_O_20_	kaempferol hexose-hexose deoxyhexose	611.1576/449.4065/287.0547	2	[19]
**24**	18.63	756.2112	757.2192	C_33_H_40_O_20_	kaempferol hexose hexose deoxyhexose	611.1576/449.4065/287.0547	2	[19]
**25**	19.59	491.3094	492.3173	C_24_H_46_O_9_N	morusimic acid C	330.2640	3	[18]
**26**	21.39	610.1534	611.1606	C_27_H_30_O_16_	quercetin-3-*O*-rutinoside	303.0495/465.1019	1	[25]
**27**	22.06	329.2566	330.2645	C_18_H_35_NO_4_	morusimic acid B	312.2529/268.2630/250.2525	3	[18]
**28**	22.42	464.0954	465.1027	C_21_H_20_O_12_	quercetin-hexoside	303.0498	2	[17]
**29**	24.39	594.1584	595.1664	C_27_H_30_O_15_	kaempferol-3-*O*-rutinoside	449.1066/287.0546	1	[17]
**30**	24.88	550.0958	551.1038	C_24_H_22_O_15_	quercetin-malonylhexoside	303.0499	2	[26]
**31**	25.10	516.1268	517.1341	C_25_H_24_O_12_	dicaffeoylquinic acid I	163.0387	2	[27]
**32**	25.59	448.1006	449.1078	C_21_H_20_O_11_	kaempferol-hexoside	287.0546	2	[17]
**33**	25.66	516.1268	517.1341	C_25_H_24_O_12_	dicaffeoylquinic acid II	325.0913/163.0387	2	[27]
**34**	27.83	516.1268	517.1341	C_25_H_24_O_12_	dicaffeoylquinic acid III	325.0913/163.0387	2	[27]
**35**	28.53	534.1009	535.1088	C_24_H_22_O_14_	kaempferol malonyl hexoside	287.0546	2	[28]

**Table 2:** Retention time window of bioactive 96-well fractions and putatively corresponding compounds (numbers refer to Table 1) in *M. alba* and *M. nigra*. n.i. = not identified.

*M. alba* Retention Time (min)	Bioactive Metabolite	*M. nigra* Retention Time (min)	Bioactive Metabolite
1.6–2.08	**1**-**2**-**3**	1.8–2.27	**1**-**2**-**3**
4.88–5.35	n.i.	4.13–4.6	n.i.
12.82–13.28	**15**	8.33–8.8	**6**
13.75–14.23	**16**/**17**	10.2–10.67	**9**
19.82–20.3	**25**	10.2–10.67	**9**
22.62–23.08	**28**	12.53–13	**15**
23.55–24.02	**29**	13.4–13.93	**16**
24.95–25.42	**32**	14.4–14.87	**17**
31.02–31.5	n.i.	15.8–16.27	**19**
34.7–35.2	n.i.	16.27–16.73	**20**
35.22–35.68	n.i.	24.67–25.12	**31**/**32**
-		40.07–40.53	n.i.

**Table 3:** Antioxidant capacity of *Morus alba* and *Morus nigra* fruits compared with strawberry fruits. Data represent average values ± standard deviation of three independent extractions. All antioxidant values are expressed as mg Trolox per g of fresh weight. TEAC: Trolox-equivalent antioxidant capacity.

Extracts	TEAC mg/g FW
*Morus alba* (White mulberry)	39.40 ± 0.02
*Morus nigra* (Black mulberry)	49.42 ± 0.01
*Fragaria vesca* (Wild strawberry)	50.61 ± 0.01
*Fragaria ananassa* (Strawberry)	51.31 ± 0.01

## Supplementary Materials

The following are available online at https://www.mdpi.com/2218-1989/10/1/7/s1, Figure S1: Representative LC-MS chromatograms, recorded in ESI positive ionization mode, of *M. nigra* (upper trace) and *M. alba* (lower trace) fruit extracts; Y-axes are on the same scale (1.5*E7; base peak intensity in ion counts/sec). Values above peaks indicate retention times (in minutes) and detected *m*/*z* value, Figure S2: Representative LC-PDA chromatograms of aqueous-methanol extracts from ripe fruits of *M. nigra* (A and B) and *M. alba* (C and D). Figures show absorbance at 520 nm (A and C) representing elution profile of anthocyanins, and at 355 nm (B and D) representing mainly flavonoids and phenylpropanoids, Values above peaks indicate retention times (in minutes). Note: intensity scales (Y-axes) are similar for all traces, Figure S3: 3 dimensional PCA plot of 5 *Morus alba* and 4 *M. nigra* fruit samples, harvested from trees spread over region Campania, Italy, based on their variation in 371 metabolites detected by the untargeted LCMS approach. The 3 quality control samples are technical replicates of a mix of samples. The X-axis (PC1) explains 33.2% of the total metabolites variation, the Y-axis (PC2) 18.6% and the Z-axis (PC3) 14.2%, Table S1: Relative intensity of all putative metabolite features (clusterID’s) for each of the analyzed mulberry samples, Table S2: Description of column heads, Table S3: MSI Identification level.
