# Supplementary material for: Identification of Bioactive Phytochemicals in Mulberries
Source: Metabolites. 2019 Dec 20;10(1):7. doi: 10.3390/metabo10010007 (PMC7023076; doi:10.3390/metabo10010007)
Supplement: Supplementary file 1 [file metabolites-10-00007-s001.zip › supplementary/supplementary materials.pdf]

metabolites

Article

Identification of Bioactive Phytochemicals in Mulberries

Gilda D'Urso [1], Jurriaan J. Mes [2], Paola Montoro [1], Robert D. Hall [3,4] and Ric C.H. de Vos [3,*]

[1] Department of Pharmacy, University of Salerno, 84084 Fisciano SA, Italy
[2] Business Unit Fresh Food and Chains, Wageningen Food & Biobased Research, Wageningen University and Research, 6708 WG Wageningen, The Netherlands
[3] Business Unit Bioscience, Wageningen Plant Research, Wageningen University and Research, 6708 PB Wageningen, The Netherlands
[4] Laboratory of Plant Physiology, Wageningen University and Research, 6708 PB Wageningen, The Netherlands
* Correspondence:ric.devos@wur.nl; Tel.: +31-317480841

Supplementary materials:

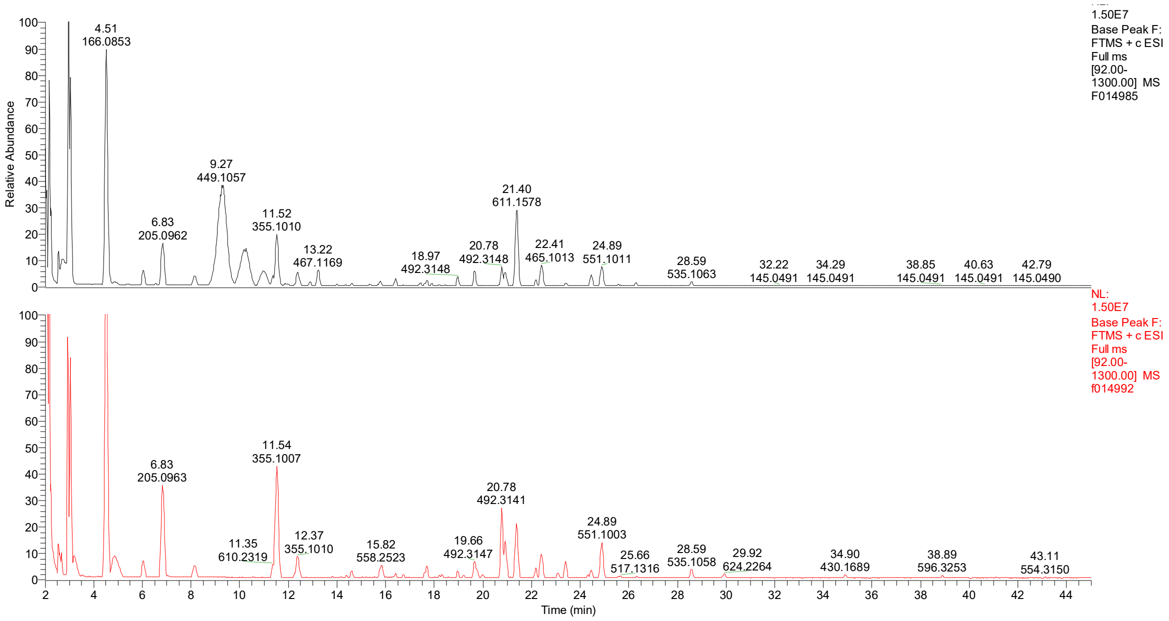

Figure S1. Representative LC-MS chromatograms, recorded in ESI positive ionization mode, of M. nigra (upper trace) and M. alba (lower trace) fruit extracts; Y-axes are on the same scale (1.5*E7; base peak intensity in ion counts/sec). Values above peaks indicate retention times (in minutes) and detected m/z value.

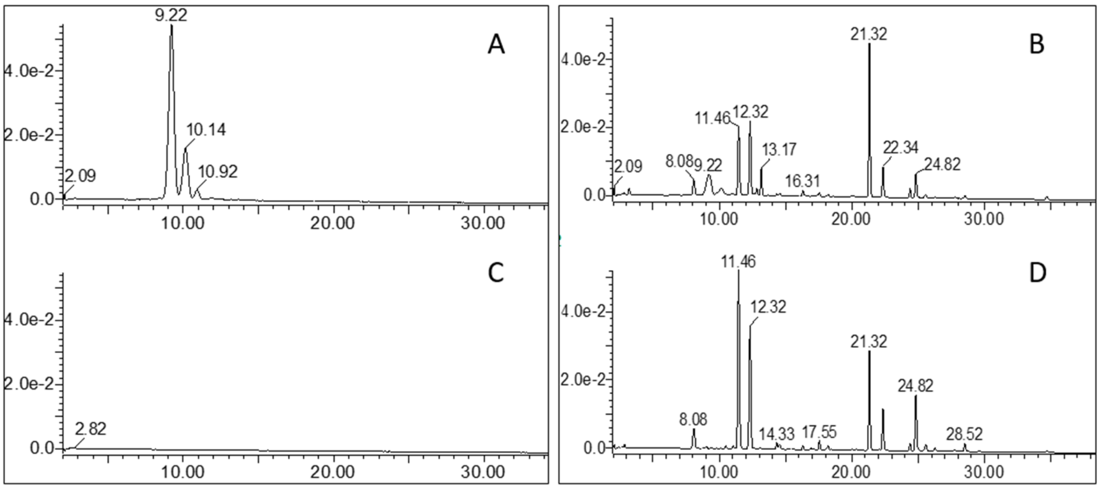

Figure S2. Representative LC-PDA chromatograms of aqueous-methanol extracts from ripe fruits of M. nigra (A and B) and M. alba (C and D). Figures show absorbance at 520nm (A and C) representing elution profile of anthocyanins, and at 355 nm (B and D) representing mainly flavonoids and phenylpropanoids, Values above peaks indicate retention times (in minutes). Note: intensity scales (Y-axes) are similar for all traces.

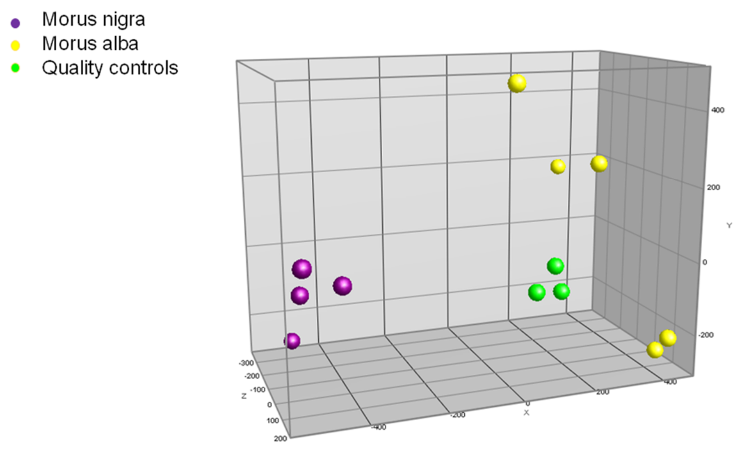

Figure S3. 3 dimensional PCA plot of 5 Morus alba and 4 M. nigra fruit samples, harvested from trees spread over region Campania, Italy, based on their variation in 371 metabolites detected by the untargeted LCMS approach. The 3 quality control samples are technical replicates of a mix of samples. The X-axis (PC1) explains 33.2% of the total metabolites variation, the Y-axis (PC2) 18.6% and the Z-axis (PC3) 14.2%.
